# AI-driven cybersecurity framework for anomaly detection in power systems

**DOI:** 10.1038/s41598-025-19634-y

**Published:** 2025-10-10

**Authors:** Vignes V. M., Sri Harini M. P., Rahul Satheesh, Vipin Das, Sanjeevikumar Padmanaban

**Affiliations:** 1 Amrita School of Artificial Intelligence, Amrita Vishwa Vidyapeetham, Coimbatore, Tamil Nadu India; 2 Department of Electrical Engineering, IT and Cybernetic, University of South-Eastern Norway, Porsgrunn, Norway

**Keywords:** Cybersecurity, Smart grid, LSTM, Random forest, SHAP, Intrusion detection system, Data fusion, Real-world implementation, Adversal analysis, Engineering, Mathematics and computing

## Abstract

The rapid evolution of smart grid infrastructure, powered by the integration of IoT and automation technologies, has simultaneously amplified the sophistication and frequency of cyber threats. Critical vulnerabilities such as False Data Injection Attacks (FDIA), Denial-of-Service (DoS), and Man-in-the-Middle (MiTM) attacks pose significant risks to the reliable and secure operation of power systems. Traditional rule-based security mechanisms are increasingly inadequate, lacking both contextual awareness and real-time adaptability. This paper introduces a precision-engineered AI-driven cybersecurity framework that fuses cyber and physical datasets to enable high-accuracy anomaly detection in power systems. Leveraging Long Short-Term Memory (LSTM) networks and Random Forest classifiers, the proposed framework achieves 99.798% accuracy in binary classification and 98.1919% accuracy in multi-class classification tasks. Interpretability is enhanced through SHapley Additive exPlanations (SHAP), which clarify feature contributions to model predictions. Robustness against adversarial threats is achieved using Fast Gradient Sign Method (FGSM)-based adversarial training, improving adversarial accuracy from 95.15 to 99.39%. The system’s practical viability is demonstrated via deployment on the Xilinx PYNQ-Z2 edge device, completing model inference in just 2.16 seconds. These results underscore the framework’s efficacy, explainability, and operational resilience, making it well-suited for real-time deployment in smart grid environments.

**Supplementary Information:**

The online version contains supplementary material available at 10.1038/s41598-025-19634-y.

## Introduction

Modern power systems have evolved into complex cyber-physical ecosystems, interweaving operational technologies (OT) such as Supervisory Control and Data Acquisition (SCADA) systems with Information and Communication Technologies (ICT). While this convergence enhances system efficiency, scalability, and responsiveness, it also introduces a broader attack surface vulnerable to increasingly sophisticated cyber threats. Among the most critical of these are False Data Injection Attacks (FDIA), where adversaries manipulate sensor data or state estimators to mislead grid operators or automated controls without triggering alarms. In Denial of Service (DoS) attacks, the goal is to disrupt communication channels or overload system resources, causing delays or blackouts. Man-in-the-Middle (MiTM) attacks compromise the integrity and confidentiality of communications by intercepting and altering messages between control centers and field devices. These attacks can result in physical damage, economic losses, and cascading failures across the grid. Their stealthy and adaptive nature often allows them to evade traditional rule-based Intrusion Detection Systems (IDS), particularly when launched as zero-day attacks with no known signature. This escalating threat landscape necessitates intelligent, adaptive, and explainable cybersecurity mechanisms capable of real-time detection and response across both cyber and physical domains. Recent frameworks such as Grid Sentinel^1^ demonstrate the effectiveness of LSTM-based models in detecting anomalies across cyber-physical layers in real-time smart grid operations.

The increasing integration of IoT, smart grid infrastructure, and automation in power systems has brought significant improvements in efficiency and control. However, these advancements have also exposed critical vulnerabilities in cyber-physical systems (CPS). Cyber threats such as FDIA, DoS, and MiTM attacks pose a severe risk to the reliable and secure operation of power grids^2,3^. Traditional rule-based cybersecurity systems often lack the real-time responsiveness, explainability^4^, and resilience required to defend against these sophisticated threats, particularly zero-day exploits that evade signature-based detection^5,6^. To overcome these limitations, an AI-Driven Cybersecurity Framework is proposed that leverages multi-source data fusion and machine learning (ML)/deep learning (DL) techniques to detect anomalies and intrusions in power systems. By fusing data from both the cyber and physical domains^7^, the system captures correlations that are otherwise undetectable when each domain is analyzed in isolation.

The framework enhances detection accuracy and reduces false positives by employing supervised and unsupervised models, explainable AI (SHAP), and a user-friendly dashboard interface. This approach builds upon the findings of Sahu et al.^7^, which demonstrated that integrating information from diverse sensors and domains significantly improves intrusion detection. The proposed solution further extends this by including state-of-the-art DL models like LSTM and GRU, comprehensive preprocessing techniques, and multi-class classification capabilities to differentiate between various types of attacks. The authors in^8^ highlight the trade-offs between accuracy, computational cost, and energy efficiency, particularly in edge environments. This motivates the choice of lightweight yet effective models like Random Forest for real-time smart grid deployment.

**Table 1:** Comparison of related works on cybersecurity in power systems.

Paper	Year	Key contribution	Methodology	Research gap
^7^	2021	Demonstrated improved IDS accuracy using fused cyber-physical data	Centralized data fusion + supervised, unsupervised ML models	Focused on binary classification but lacks multi-class labelling of various attack types and limited integration of advanced DL models like LSTM/GRU for sequential learning
^9^	2021	Shows CNN effectiveness for binary anomaly detection	Deep learning (CNN) on SCADA data	Handles only binary classification doesn’t differentiate attack types and trained only on cyber-side data, no physical measurement integration and lacks explainability
^10^	2022	Showed LSTM and GRU perform well in anomaly detection	Recurrent neural networks (RNNs) with labeled data	No interpretability tools to understand why an alert was triggered and no visualization or operational interface for monitoring anomalies
^11^	2021	Two-stage supervised ML with pre-processing	Used layered ML for step-wise anomaly classification	Sequential learning used, but without physical sensor fusion and no deep learning integration for long-term dependencies
^5^	2024	Benchmarked GNN against classic ML in power system IDS	Performance comparison using labeled datasets	Limited validation on multi-class attack detection or fused cyber-physical datasets

Sahu et al.^7^ proposed a novel framework that fuses sensor data from both cyber (e.g., packet captures, Snort alerts) and physical (e.g., DNP3 protocol-based measurements) sources. Their centralized data fusion architecture utilizes supervised, semi-supervised, and unsupervised learning algorithms to enhance detection performance. This approach addresses challenges like data synchronization, sensor heterogeneity, and differences in time resolution across sources.

As shown in the Table 1, existing studies have explored a variety of methods for detecting anomalies in smart grid environments. While frameworks like that of A. Sahu et al. introduced cyber-physical data fusion, they lacked advanced sequential modeling. Other models focused on classical ML or deep learning techniques, but often neglected explainability. Our proposed framework addresses these limitations by integrating LSTM and Random Forest models with explainability tools like SHAP for a comprehensive, interpretable, and accurate anomaly detection system.

Despite advancements in anomaly detection for cyber-physical power systems, current methods often fall short in handling sophisticated threats such as FDIA, DoS, and MiTM attacks. These attacks exploit vulnerabilities across both cyber and physical layers, often evading traditional detection mechanisms that rely on predefined signatures or isolated data sources. There remains a critical need for a unified, intelligent, and explainable framework capable of detecting both known and novel anomalies in real time.

To address the limitations identified in existing literature, this work introduces several key innovations that enhance anomaly detection in cyber-physical power systems:

1. A novel AI-based framework that fuses cyber (network traffic, Snort alerts) and physical (DNP3 protocol-level) features for anomaly detection in power systems.
2. Support for both binary and multi-class classification tasks, enabling the system to distinguish among multiple attack types.
3. Advanced deep learning models such as Long Short-Term Memory (LSTM) and Gated Recurrent Units (GRU) are employed to capture temporal dependencies in sequential system data, enhancing the detection of persistent and evolving threats. To ensure model transparency, SHAP (SHapley Additive exPlanations) is utilized, offering interpretable insights into model decisions and feature contributions.
4. Application of adversarial training using FGSM to enhance resilience against evasion attacks.
5. Deployment of the Random Forest model on a Xilinx PYNQ-Z2 edge device, validating the framework’s practicality for real-time smart grid environments.

## Dataset description

The dataset used in this study is the Cyber-Physical Dataset for MiTM Attacks in Power Systems developed by RESLab, Texas A&M University, Unites States and it is publicly available via IEEE Dataport^12^. The dataset is based on the DNP3 protocol, which is one of the most widely deployed standards in North American power grids, and therefore provides a realistic and reproducible basis for experimentation. It includes synchronized cyber and physical measurements along with well-documented attack scenarios such as False Data Injection, Command Injection, and Man-in-the-Middle attacks. While the present work is scoped to DNP3, we acknowledge that real-world power grids also incorporate protocols such as IEC 61850 and Modbus.

It is derived from a simulated grid environment using the DNP3 protocol, which captures both physical and communication-layer behaviors through structured messages. While it does not specify the explicit topology such as the number of loads, generators, or substations, it abstracts grid operations through analog values, control commands, counters, and time delays. This protocol-level abstraction reflects realistic operational behavior without depending on a fixed physical layout, enabling generalizable anomaly detection. Each DNP3 message simulates activity from typical grid components, ensuring that critical patterns of normal and anomalous behavior are preserved, even though physical quantities like the number of connected devices are not directly represented. This dataset encompasses data from both network traffic logs and power system parameters recorded during simulated cyberattacks, enabling the detection of anomalies at the intersection of IT (network) and OT (operational technology) in power systems. The dataset directories include:

- **Adversary/** This folder contains the Json files for the DNP3 and ARP based packets captured at the attacker used for constructing the labels.
- **csvs/** This folder contains all the processed and encoded files obtained after the merge and extraction process from multiple sources. The sub-directories distributes the files based on the use case.
- **RawFiles/** This folder contains raw files obtained from different sources. This folder contains sub directories : DS, master, router and snort. The snort sub-directory contains snort logs running in the substation router for different use-cases.

**Table 2:** Network and transport layer features for cybersecurity analysis.

Features	Description
Frame Length	Length of the frame after network, transport and application header and payload are added and fragmented based on the channel type.
Frame Protocols	Determines the list of protocols in the layers above link layer encapsulated in the frame.
Ethernet Source	Unique source MAC address. Crucial for detection in ARP spoof attacks.
Ethernet Destination	Unique destination MAC address. Crucial for detection in ARP spoof attacks.
IP Source	Unique source IP address.
IP Destination	Unique destination IP address.
IP Length	Stores the length of the header and payload in a IP-based packet. This correlates well with the DNP3 payload size.
IP Flags	Indicator of fragmentation caused due to link or router congestion in the intermediary nodes.
Source Port	Indicates the port number used by the source application using TCP in transport layer.
Destination Port	Indicates the port number used by the destination application using TCP in transport layer.
TCP Length	Stores the length of the header and payload in a TCP-based segment. This correlates well with the DNP3 payload size.
TCP Flags	Flags are used to indicate a particular state of connection such as SYN, ACK, etc.
Retransmission	Indicates if the current record is from a retransmitted packet, caused due to attack or network congestion.
Round Trip Time	Indicator of propagation and processing delay. High RTT can be caused due to MiTM attack.
Final Flow Count	Indicates if the current flow carries the final packet.
Packet Count	Number of packets transmitted in a specific time interval.
Snort Alert	Boolean indicating an alert from snort.
Snort Alert Type	Indicates the alert type such as DNP3, ARP spoof, ICMP flood or any other types.
DNP3 Link Layer Source	Source id of the DNP3 master or outstation. Indicator of which outstation communicates with the master in that specific record.
DNP3 Link Layer Destination	Destination id of the DNP3 master or outstation. Indicator of which outstation communicates with the master in that specific record.
DNP3 Link Layer Length	Indicator of the DNP3 payload size as well as the function type.
DNP3 Link Layer Control Flag	This indicates the initiator of the communication. Determines the primary/secondary server.
DNP3 Transport Layer Control Flag	Indicates the FIN/FIR/Sequence number for determining if the DNP3 payload is the first or final segment.
Function Code	Indicates the function code: either READ, WRITE, OPERATE, DIRECT OPERATE, etc.
Application Layer Control Flag	Indicates the FIN/FIR/Seq/Confirm and Unsolicited flags. This indicates if there are unsolicited, first, final from application layer standpoint.
DNP3 Object Count	This count determines the number of BI, BO, AI, AO points associated with a substation.
DNP3 Application Layer Payload	Contains the DNP3 points used to extract the physical features such as branch status, real power flows and injections in branch and buses for a substation.

This comprehensive dataset facilitates anomaly detection in both IT and OT domains and supports both binary and multi-class classification tasks. The features of the dataset has been listed in the Table 2. Similar to prior work on securing GOOSE messages^13^, this framework leverages lower-layer protocol indicators for early-stage anomaly detection. The dataset defines four use cases (UC1–UC4), each corresponding to distinct categories of cyberattacks that target both the communication and physical layers of the grid:

- UC1 – False Command Injection (FCI): Relay control commands are falsified to disconnect legitimate lines or overload alternate ones, causing operational imbalance.
- UC2 – Generator Setpoint Tampering: Malicious alteration of generator setpoints (both analog and binary), leading to incorrect power dispatch and destabilization of the grid.
- UC3 – Combined FDIA + FCI: Adversaries simultaneously inject false measurement data and malicious commands, complicating detection efforts.
- UC4 – Multi-Stage Stealth Attack: A coordinated sequence involving falsified measurements, malicious command injections, and falsified confirmations, simulating persistent stealth intrusions.

These use cases are explicitly documented in the dataset^12^. They capture realistic adversarial behaviors that span both cyber and physical domains, and serve as the foundation for the anomaly detection experiments in this work. The data from all the use cases are merged into one datafile for classifying various types of attacks which consists of 2473 labeled instances, distributed as follows: UC1 – 507, UC2 – 551, UC3 – 673, UC4 – 742 samples. Each record contains fused cyber-physical features. This approach adopted an early fusion strategy, where cyber and physical features were combined into a single feature vector before model training. This approach was chosen for its simplicity and effectiveness in enabling the model to learn joint representations from both domains. Given the practical constraints of edge deployment, early fusion reduces architectural complexity and minimizes the need for separate pre-processing or sub-models for each modality. While late fusion or hierarchical approaches may offer improved flexibility or modularity, they also introduce additional computational overhead. Comparative evaluation of different fusion strategies remains a valuable direction for future research.

## Methodology

### Preprocessing

To address potential data asynchrony between cyber and physical measurements, the dataset provides a time-synchronized logging mechanism that aligns both domains using common timestamps. Each DNP3 packet or control command is tagged with the corresponding physical system state, allowing direct fusion across modalities. In cases where cyber and physical streams operate at different sampling rates, a sliding window approach was employed to aggregate events into fixed-length intervals suitable for sequential modeling. Continuous variables such as analog measurements were interpolated when missing, while discrete events such as command signals were forward-filled to maintain temporal consistency. This preprocessing ensures that both cyber and physical features remain synchronized, enabling models such as LSTM to effectively capture cross-domain temporal dependencies.

Redundant columns were removed, and missing values were imputed using either the mean or zero. Attack use cases UC1–UC4 were numerically encoded (0–3). Correlation heatmaps and distribution plots were generated to understand feature characteristics. Principal Component Analysis (PCA) was employed for dimensionality reduction to visualize class separability and detect outliers.

### PCA for dimensionality reduction

PCA is a dimensionality reduction method that transforms high-dimensional data into a lower-dimensional space while retaining the maximum variance. It begins by standardizing the data using the Eq. (1), where each feature is centered and scaled.

1 $$\begin{aligned} X_{\text {standardized}} = \frac{X - \mu }{\sigma } \end{aligned}$$

where $\mu$ and $\sigma$ are the mean and standard deviation of each feature. Next, the covariance matrix $\Sigma$ is computed as given in the Eq. (2).

2 $$\begin{aligned} \Sigma = \frac{1}{m-1} X^\top X \end{aligned}$$

capturing the relationships between features. Eigenvalues $\lambda _i$ and eigenvectors $v_i$ of $\Sigma$ are then calculated, where the eigenvectors represent the new axes (principal components) and the eigenvalues indicate the variance captured by each component. Finally, the data is projected onto the top *k* principal components using the Eq. (3).

3 $$\begin{aligned} X_{\text {PCA}} = X \cdot V_k \end{aligned}$$

where $V_k$ contains the eigenvectors corresponding to the *k* largest eigenvalues.

This transformation simplifies the dataset while preserving its most important patterns, making PCA valuable for visualization and preprocessing.

### Random forest for supervised learning

Random Forest is an ensemble-based supervised learning algorithm that operates by constructing a multitude of decision trees during training and aggregating their outputs to enhance generalization performance. The method combines both bagging and random feature selection to mitigate overfitting and improve predictive accuracy^5,14^. Given a dataset $D = \{(x_1, y_1), (x_2, y_2), \ldots , (x_n, y_n)\}$, Random Forest creates $T$ bootstrap samples $D^{(1)}, D^{(2)}, \ldots , D^{(T)}$ by sampling with replacement from $D$. Each bootstrap sample is used to train an individual decision tree. At each node of a tree, instead of evaluating all features, a random subset of $m$ features (where $m < p$, the total number of features) is selected, and the best split is determined based on an impurity criterion.

**Fig. 1:**
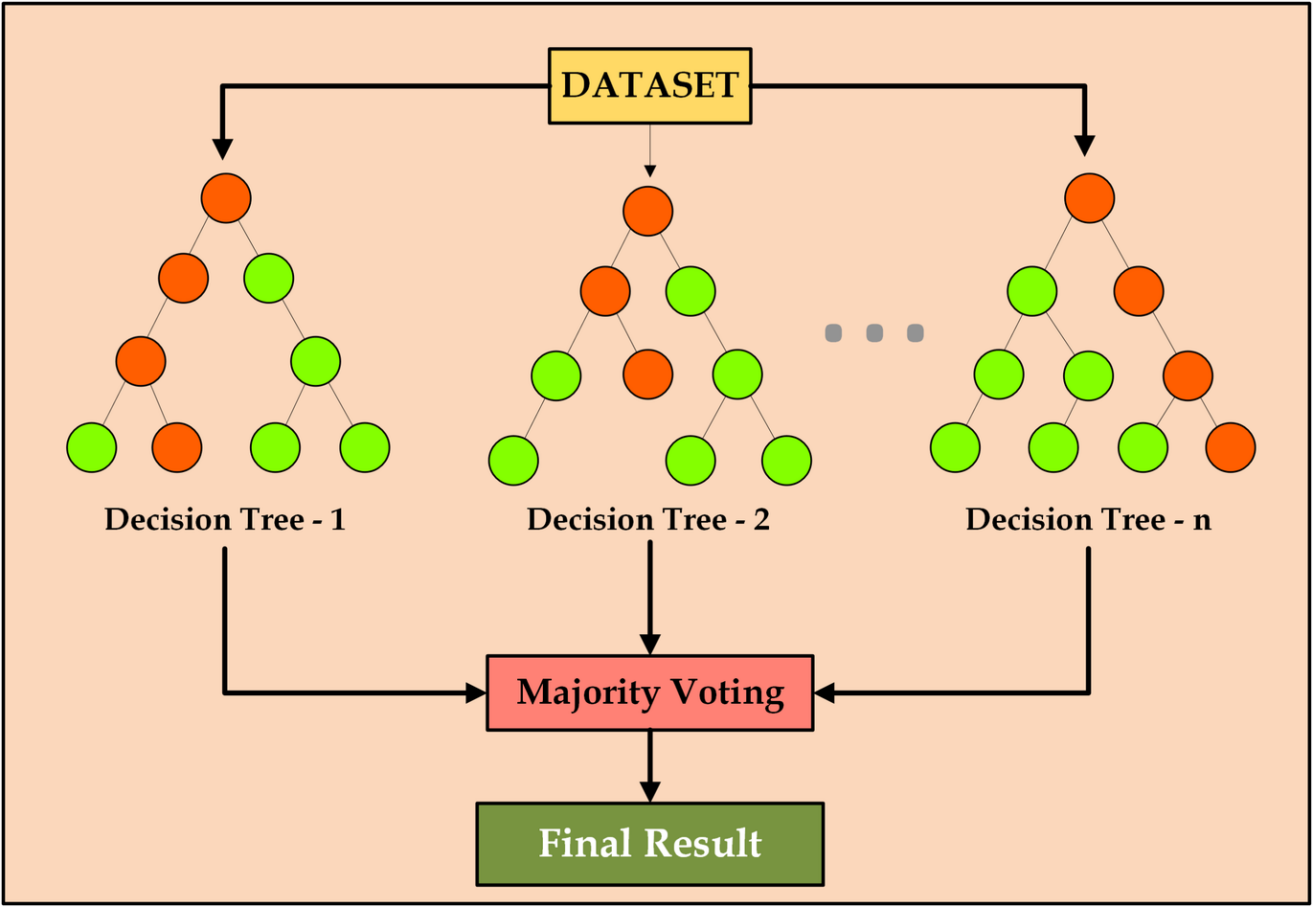
Random forest process flow.

For classification tasks, Gini impurity or entropy is commonly used to evaluate split quality. The Gini impurity is defined as given in Eq. (4).

4 $$\begin{aligned} Gini(D) = 1 - \sum _{i=1}^{C} p_i^2 \end{aligned}$$

where $p_i$ is the proportion of class $i$ instances in node $D$, and $C$ is the number of classes. Alternatively, entropy is given by Eq(5).

5 $$\begin{aligned} H(D) = - \sum _{i=1}^{C} p_i \log _2(p_i) \end{aligned}$$

The final prediction is obtained by aggregating the outputs of all decision trees. For classification, this is achieved via majority voting as per Eq. (6)

6 $$\begin{aligned} H(x) = \text {mode} \{ h_1(x), h_2(x), \ldots , h_T(x) \} \end{aligned}$$

where $h_t(x)$ is the prediction of the $t$-th tree. For regression tasks, the final output is the average of the individual predictions as per Eq. (7)

7 $$\begin{aligned} H(x) = \frac{1}{T} \sum _{t=1}^{T} h_t(x) \end{aligned}$$

An internal validation mechanism, known as Out-of-Bag (OOB) estimation, is employed to assess the generalization performance.

Since each tree is trained on approximately two-thirds of the dataset, the remaining one-third (the OOB samples) can be used to evaluate prediction error without requiring a separate validation set. Additionally, Random Forest facilitates feature importance ranking by averaging the decrease in impurity contributed by each feature across all trees (Fig. 1). This capability enhances the model’s interpretability and guides feature selection in high-dimensional datasets.

### LSTM model for supervised learning

**Fig. 2:**
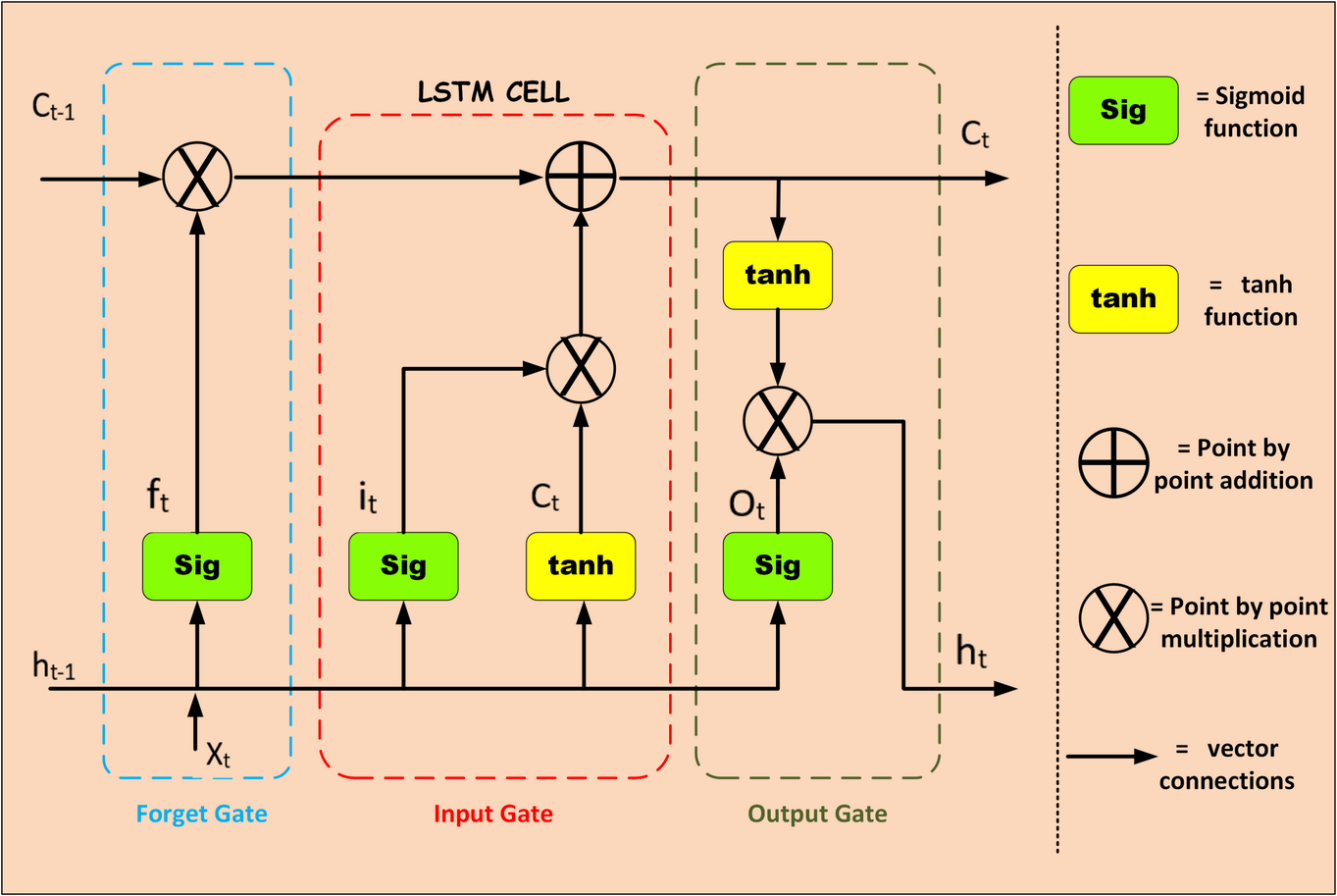
LSTM Architecture.

LSTM networks are a specialized form of Recurrent Neural Networks (RNNs) designed to capture long-range dependencies in sequential data by addressing the vanishing gradient problem commonly encountered in standard RNNs^10,15,16^. An LSTM unit comprises a cell state and three gating mechanisms: the input gate, forget gate, and output gate, which together regulate the flow of information through the sequence (Fig. 2).

Let $x_t \in \mathbb {R}^{n}$ be the input vector at time step $t$, $h_{t-1} \in \mathbb {R}^{m}$ be the hidden state from the previous time step, and $C_{t-1} \in \mathbb {R}^{m}$ be the previous cell state. The operations of an LSTM unit are defined by the following equations:

#### Forget gate

The forget gate $f_t$ determines which parts of the previous cell state $C_{t-1}$ should be retained:

8 $$\begin{aligned} f_t = \sigma (W_f x_t + U_f h_{t-1} + b_f) \end{aligned}$$

#### Input gate

The input gate $i_t$ decides which new information should be stored in the cell:

9 $$\begin{aligned} i_t = \sigma (W_i x_t + U_i h_{t-1} + b_i) \end{aligned}$$

#### Candidate cell state

The candidate cell state $\tilde{C}_t$ holds potential new content for the memory:

10 $$\begin{aligned} \tilde{C}_t = \tanh (W_c x_t + U_c h_{t-1} + b_c) \end{aligned}$$

#### Cell state update

The cell state $C_t$ is updated by combining retained memory and new candidate values:

11 $$\begin{aligned} C_t = f_t \odot C_{t-1} + i_t \odot \tilde{C}_t \end{aligned}$$

#### Output gate

The output gate $o_t$ controls the information passed to the next hidden state:

12 $$\begin{aligned} o_t = \sigma (W_o x_t + U_o h_{t-1} + b_o) \end{aligned}$$

#### Hidden state

Finally, the hidden state $h_t$, serving as both output and feedback, is:

13 $$\begin{aligned} h_t = o_t \odot \tanh (C_t) \end{aligned}$$

The forget gate (Eq. (8)) decides what to retain from the past. The input gate (Eq. 9) and candidate state (Eq. 10) inject new information. The cell state update (Eq. 11) merges old and new memory. The output gate (Eq. 12) and updated cell state regulate the final hidden state (Eq. 13).

LSTM networks are particularly effective in modeling temporal dependencies in time series data, natural language, and sequences in general, making them suitable for applications such as forecasting, classification, and signal processing.

### SHAP explainability for model interpretability

SHAP (SHapley Additive exPlanations) values provide a game-theory-based approach to attribute the contribution of each feature to the prediction of a machine learning model^4,17,18^. Recent efforts on interpretable fault detection align with the integration of SHAP for decision transparency in anomaly prediction^19^. For a model $f(x)$, SHAP values fairly distribute the prediction across the features, ensuring that their sum equals the model’s output.

**Fig. 3:**
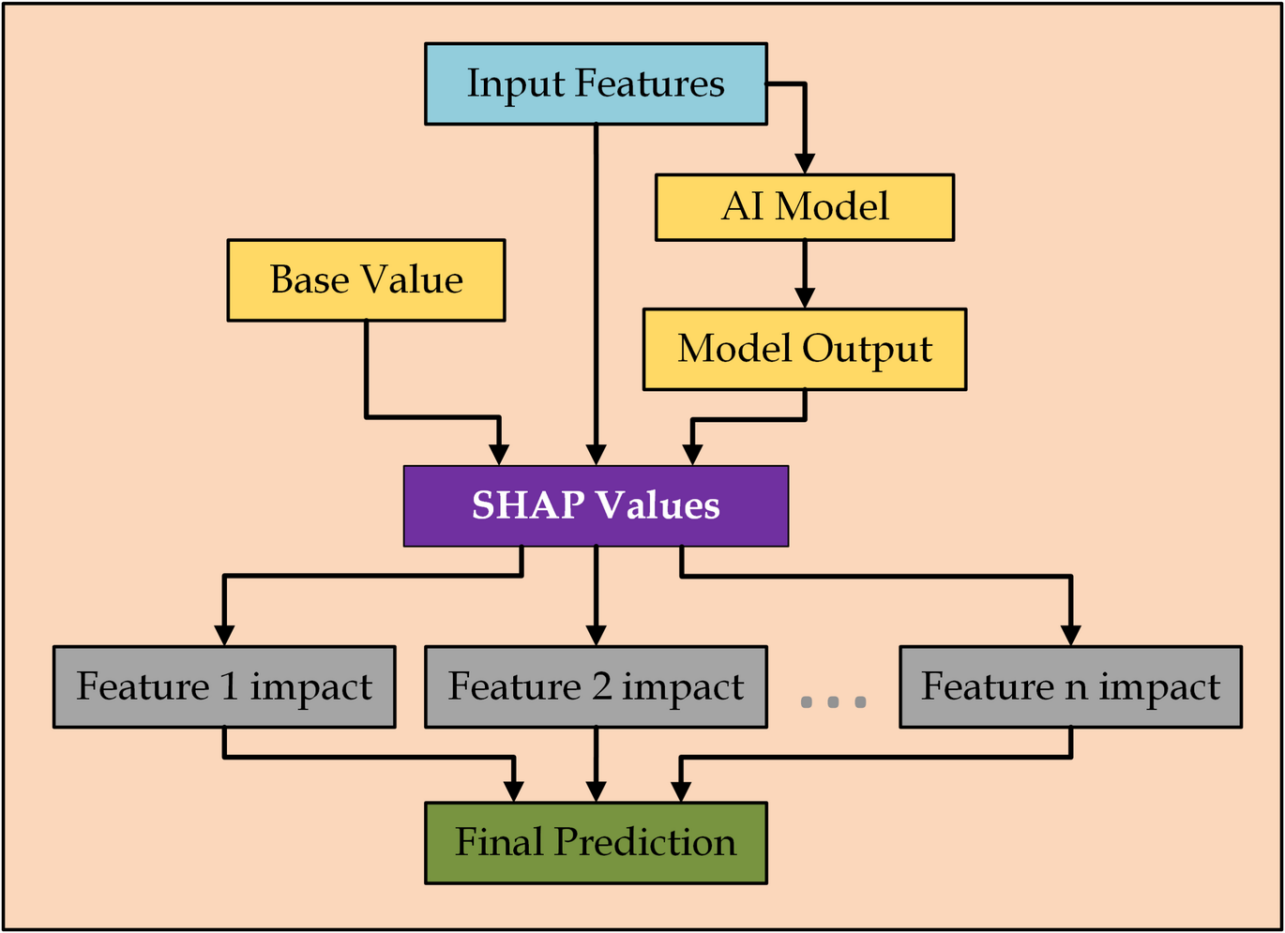
SHAP process flow.

The Shapley value for feature $x_i$ is calculated as,

$$\phi _i(f) = \sum _{S \subseteq N \setminus \{i\}} \frac{|S|!(|N|-|S|-1)!}{|N|!} \left[ f(S \cup \{i\}) - f(S) \right]$$

where $S$ is a subset of features excluding $x_i$, and $f(S)$ is the model’s prediction with features in subset $S$. This measures the marginal contribution of $x_i$ averaged over all possible feature subsets.

To approximate the Shapley value, Monte Carlo sampling can be used,

$$\hat{\phi }_i(f) \approx \frac{1}{M} \sum _{m=1}^{M} \left[ f(S_m \cup \{i\}) - f(S_m) \right]$$

where $S_m$ is a random subset of features.

SHAP values satisfy several key properties: efficiency (the sum of all feature contributions equals the prediction), symmetry (features with equal contributions have the same value), and linearity (the Shapley values of combined models equal the sum of individual values). SHAP values enable transparent and interpretable machine learning by quantifying feature importance in both classification and regression tasks.

For example, in a classification model, SHAP values help identify which features push the decision towards a certain class, making the model’s predictions interpretable (Fig. 3).

## Results and discussion

This section presents the outcomes of our experiments and provides insights into the performance of the proposed AI-driven cybersecurity framework for anomaly detection in power systems. The models were evaluated using a fused cyber-physical dataset comprising network traffic, DNP3 logs, and operational power parameters. The discussion covers feature analysis, model performance in both binary and multi-class classification tasks, and explainability insights using SHAP values.

### Feature correlation

**Fig. 4:**
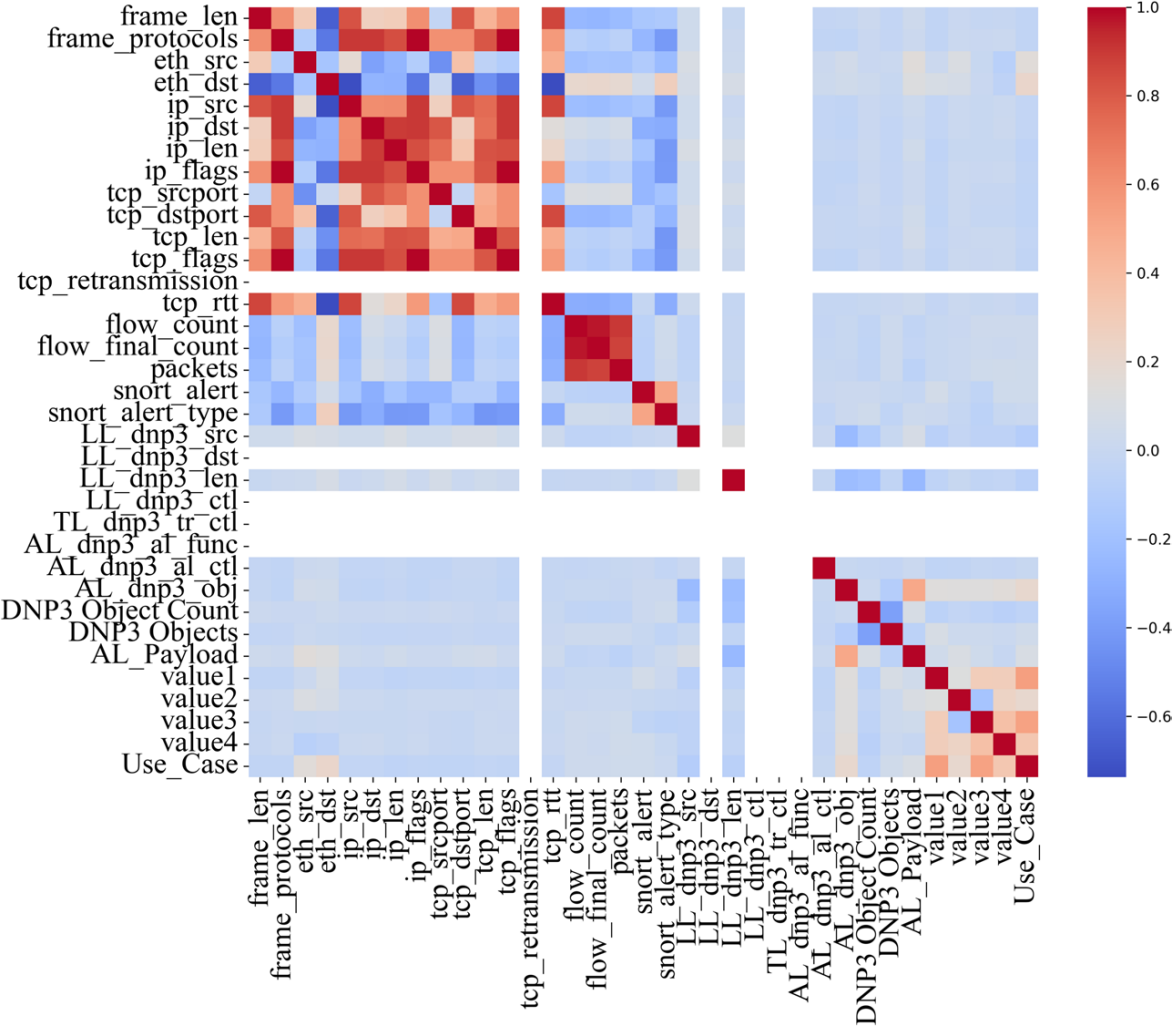
Feature correlation heatmap showing dependencies among cyber and physical features.

A correlation heatmap (Fig. 4) was generated to understand the relationships among features. Features related to the network layer-such as frame_protocols, eth_dst, ip_dst, and tcp_flags exhibited strong mutual correlations.

**Fig. 5:**
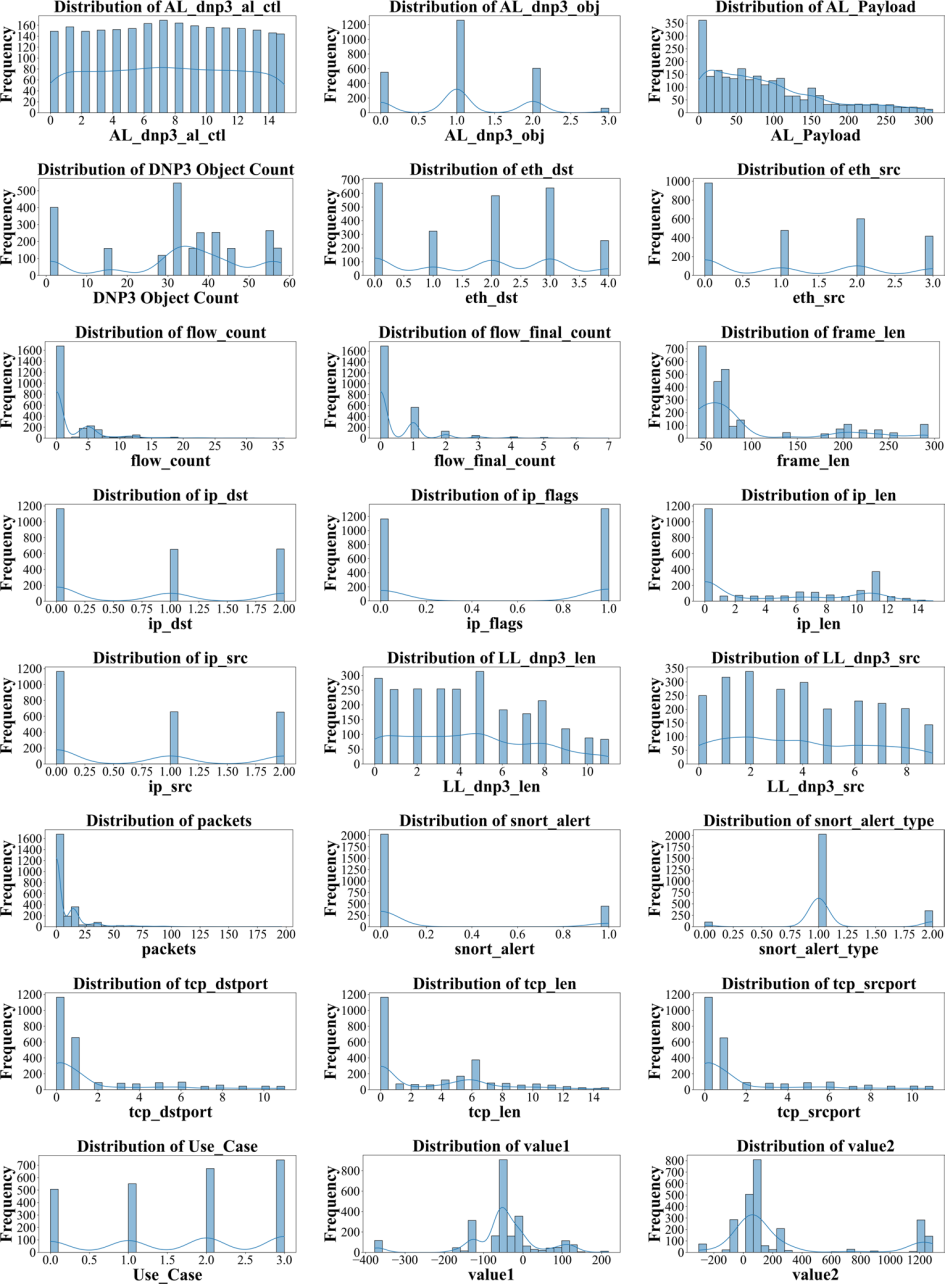
Data distribution of several features.

In contrast, features such as snort_alert, LL_dnp3_src, and various DNP3 protocol measurements showed minimal correlation with other features. While network-layer features tend to cluster, DNP3-specific parameters exhibit statistical independence^7,20^, suggesting they offer unique signals for anomaly detection. This validates their inclusion in the fused dataset for robust model learning.

### Data distribution

In terms of data distribution (Fig. 5), several features demonstrated skewed distributions or binary-like characteristics (e.g., snort_alert, tcp_retransmission). Others, like frame_len and AL_Payload, presented continuous distributions with outliers, potentially signifying anomalous or malicious activity.

The diverse data distributions justify the use of both deep learning (for sequential, non-linear patterns) and ensemble models (for feature-rich tabular data).

### Feature importance and dimensionality reduction

**Fig. 6:**
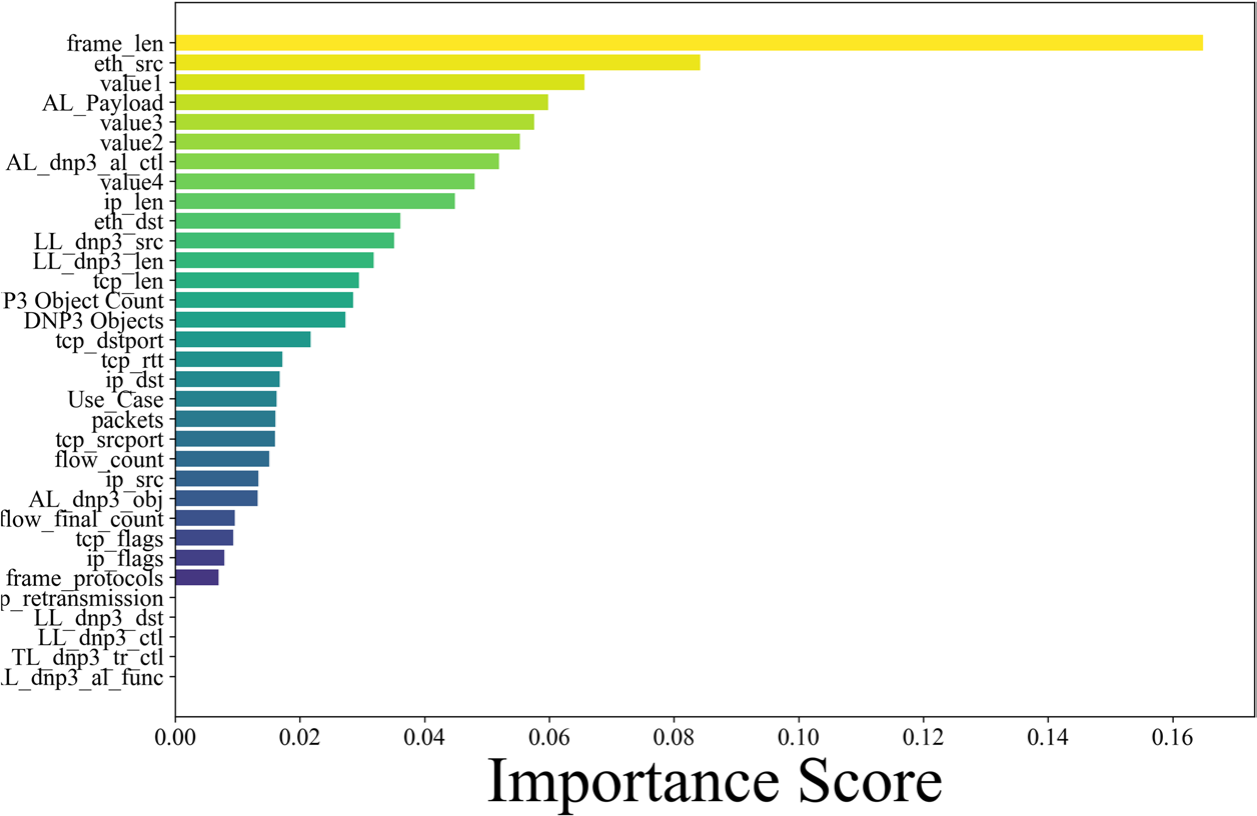
Feature importance using random forest.

Feature importance derived from Random Forest (Fig. 6) showed frame_len as the most critical predictor of snort_alert, followed by value1–value4, AL_Payload, and MAC address fields (eth_src, eth_dst)^14^. These findings suggest that alert mechanisms are sensitive not only to metadata but also to content-level anomalies.

**Fig. 7:**
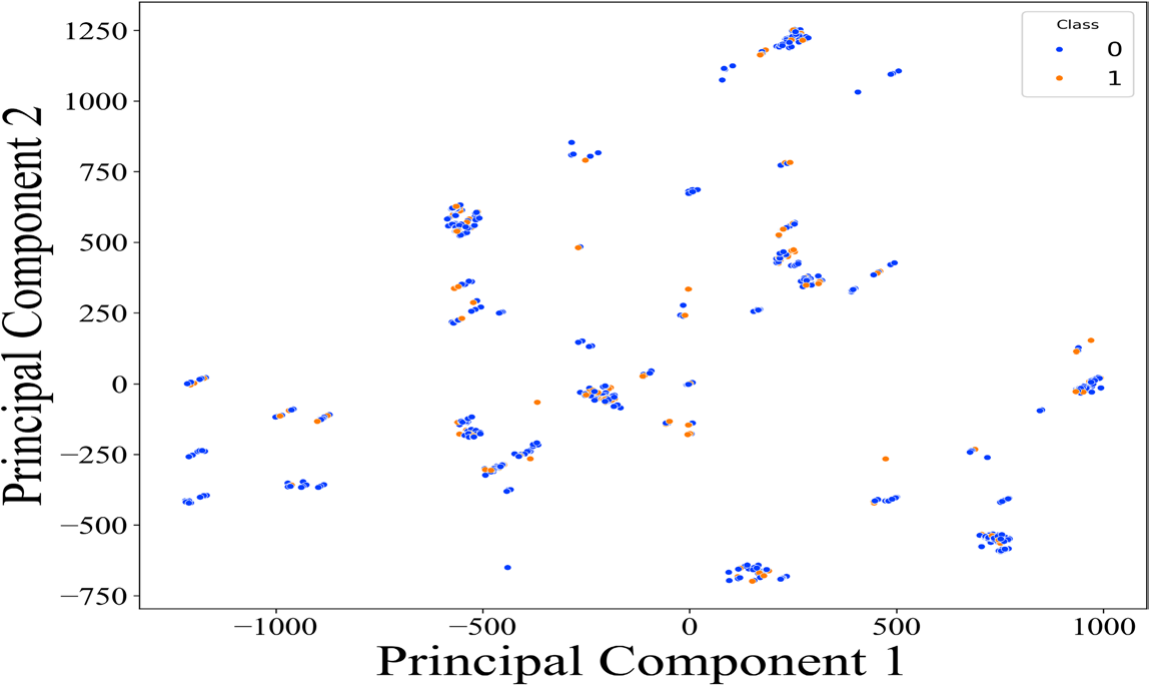
Dimensionality reduction using PCA.

Dimensionality reduction using PCA (Fig. 7) revealed one significant outlier, while the rest of the dataset exhibited tight clustering implying low variance in normal operations. The lack of clear separability also indicated that linear models may be suboptimal for this problem space.

The prominence of both payload and identity-related features in driving anomalies supports the need for hybrid feature modeling. PCA also provides visual confirmation of rare anomalies and potential noise. This make the dataset to be ready for the model training, the input features to the models span both cyber and physical domains. From the cyber side, features include packet sizes (frame_len, ip_len), protocol flags (tcp_flags, tcp_rtt), and Snort alert indicators. Physical features are extracted from the DNP3 application and transport layers, such as DNP3 Object Count, value1–value4, and AL_Payload, which correspond to real power flows, breaker status, and injection levels in substations.

The model outputs a classification label, either binary (normal vs. attack) or multi-class (UC1–UC4), identifying the nature of the detected anomaly.

### Binary classification performance

**Fig. 8:**
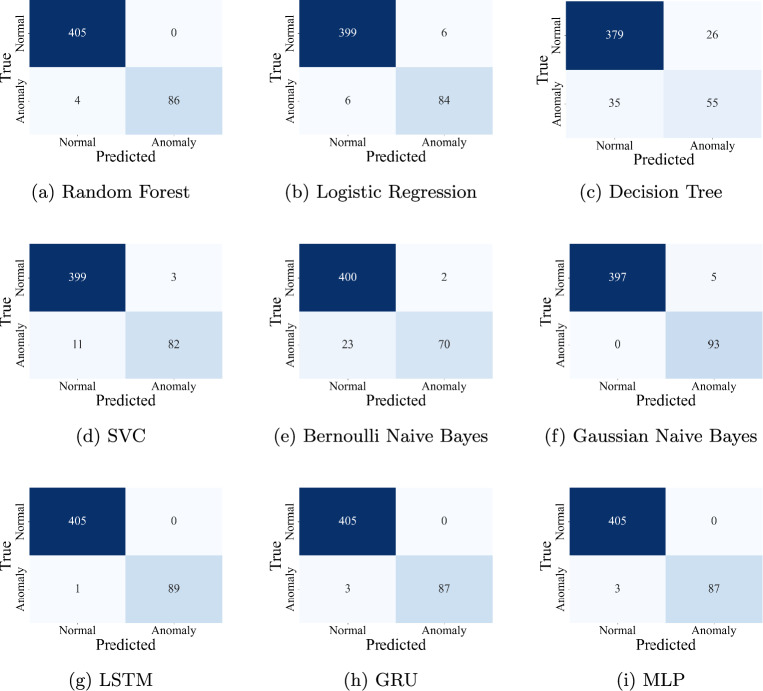
Confusion matrices of binary classification models.

Binary classification was performed to differentiate between normal operation and anomalous behavior in the power system environment. Both traditional machine learning and deep learning models were applied to the cleaned, preprocessed dataset. The hyperparameters for all the models were selected using grid search with multiple configurations, and the best-performing combinations were retained to reduce repeated compilation time during further experimentation. The results of the models are shown as a confusion matrix in Fig. 8.

#### Machine learning models

A range of classical classifiers were trained and evaluated. Table 3 summarizes their performance.

**Table 3:** Binary classification accuracy - machine learning models.

Model	Accuracy	Precision	Recall	F1-Score	Confusion matrix
Random forest	**0.9919**	**1.0000**	**0.9556**	**0.9773**	Fig. 8a
Logistic regression	0.9758	0.9333	0.9333	0.9333	Fig. 8b
Decision tree	0.8768	0.6790	0.6111	0.6433	Fig. 8c
Support vector classifier	0.9717	0.9647	0.8817	0.9213	Fig. 8d
Bernoulli Naive Bayes	0.9495	0.9722	0.7527	0.8485	Fig. 8e
Gaussian Naive Bayes	0.9899	0.9490	1.0000	0.9738	Fig. 8f

Significant values are in [bold].

Among machine learning models, Random Forest achieved the highest accuracy of 99.19%. Its ensemble-based nature allowed it to efficiently capture feature interactions and reduce overfitting. Linear models such as Logistic Regression performed reasonably well but showed limitations when dealing with complex data structures. Naive Bayes variants lagged slightly, possibly due to their strong independence assumptions.

#### Deep learning models

The following deep learning models were evaluated for binary classification. Their performance is summarized in Table 4.

**Table 4:** Binary classification accuracy - deep learning models.

Model	Accuracy	Precision	Recall	F1-Score	Confusion matrix
LSTM	**0.9980**	**1.0000**	**0.9889**	**0.9944**	Fig. 8g
GRU	0.9939	1.0000	0.9667	0.9831	Fig. 8h
MLP	0.9939	1.0000	0.9667	0.9831	Fig. 8i

Significant values are in [bold].

The Long Short-Term Memory (LSTM) network outperformed all other models, achieving a remarkable accuracy of 99.7980%. This can be attributed to LSTM’s ability to capture sequential dependencies in time-series data, which are common in cyber-physical systems. GRU, a simplified variant of LSTM, also performed well and offered faster training. The Multilayer Perceptron (MLP), while not sequential, still captured non-linear patterns effectively.

Deep learning models, especially LSTM, demonstrated superior accuracy in binary classification due to their ability to model time-dependent behaviors. While Random Forest performed admirably and offered faster inference, LSTM is better suited for real-time detection in dynamic environments. The results validate the effectiveness of sequence-aware architectures in detecting cyber-physical anomalies.

### Multi-class classification performance

**Fig. 9:**
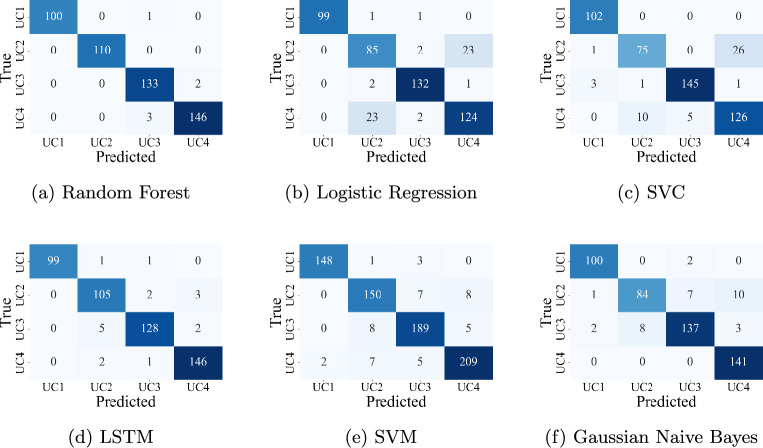
Confusion matrices of multi-class classification models.

To evaluate the system’s ability to differentiate between multiple cyberattack types, multi-class classification experiments were conducted. Modeling the reliability of subsystems under typed cyber-physical attacks provides a foundational perspective on classification resilience^21^. The models were trained to classify instances into one of several attack categories based on fused cyber-physical feature sets. The multi-class classification task included four use cases (UC1–UC4), with class counts ranging from 507 to 742. Although this represents a mild class imbalance, no explicit resampling or class weighting was applied. The models demonstrated high accuracy without requiring additional balancing techniques, likely due to the robustness of Random Forest and deep learning models in handling moderate skew. The results of the models are shown as a confusion matrix in Fig.  9.

#### Machine learning models

The following machine learning models were applied for multi-class classification. Table 5 presents their classification accuracy.

**Table 5:** Multi-class classification accuracy - machine learning models.

Model	Accuracy	Precision	Recall	F1-Score	Confusion matrix
Random forest	**0.9819**	**0.9840**	**0.9822**	**0.9830**	Fig. 9a
Support vector classifier	0.9069	0.9087	0.9005	0.9025	Fig. 9c
Logistic regression	0.8889	0.8918	0.8907	0.8912	Fig. 9b
Gaussian Naive Bayes	0.9409	0.9480	0.9365	0.9401	Fig. 9f
Support vector machine	0.9547	0.9598	0.9539	0.9563	Fig. 9e

Significant values are in [bold].

Random Forest achieved the highest classification accuracy of 98.1919%, effectively identifying different attack types with minimal confusion. Its ensemble-based decision trees and inherent handling of feature interactions contributed to superior generalization. Other models such as SVC and Logistic Regression performed well but were more prone to confusion among closely related classes. Gaussian Naive Bayes performed the weakest, likely due to the complexity and interdependence of the features.

#### Deep learning models

The deep learning models evaluated for multi-class classification, the LSTM network yielded the best results. LSTM maintained high performance with an accuracy of 97.1717%, (Fig. 9d) demonstrating strong temporal learning capabilities. However, its slight underperformance compared to Random Forest in this context may be due to class imbalance or overlapping feature patterns, which ensemble methods handle more robustly.

In multi-class settings, the Random Forest model outperforms deep learning models in terms of accuracy and class separation. Its ensemble nature makes it resilient to overfitting and class imbalance. While LSTM remains a powerful option for sequential learning, its advantage is more pronounced in binary classification. For scenarios requiring precise identification of diverse attack types, Random Forest emerges as the most reliable model.

#### Classification results

The binary classification task was trained using binary_crossentropy loss with a sigmoid output layer, while the multi-class classification used categorical_crossentropy loss along with a softmax output layer to predict the class probabilities across four use cases. This difference ensures the loss function appropriately penalizes misclassifications based on task type. For Random Forest, the Gini impurity criterion was consistently applied for both binary and multi-class setups, as it inherently adapts to the number of classes during tree construction. For LSTM, the optimal setup included the Adam optimizer with a learning rate of 0.001, a batch size of 32, and two LSTM layers with 64 units each. The model was trained for 20 epochs with dropout regularization set to 0.3. For Random Forest, grid search determined that 100 estimators (n_estimators) with the Gini impurity criterion yielded the best performance. The max_depth was left unrestricted to allow full tree growth, which enhanced class separability. These configurations were selected based on validation accuracy and suitability for deployment on edge hardware.

### Adversarial robustness performance

Resilience-focused models in DC microgrids further highlight the vulnerability of distributed systems under cyberattack scenarios^22^. Adversarial robustness was evaluated by subjecting the LSTM model to adversarial samples generated using the FGSM^16,23^. These samples, perturbed with a magnitude ($\epsilon$) of 0.02, simulated potential adversarial attacks on the IDS. An adversarially trained LSTM model was trained with a combination of clean and adversarial samples to mitigate these perturbations and improve robustness. The clean-trained LSTM model achieved a clean data accuracy of 99.80%. However, under adversarial conditions, its accuracy dropped to 95.15%, highlighting its vulnerability to adversarial attacks.

**Fig. 10:**
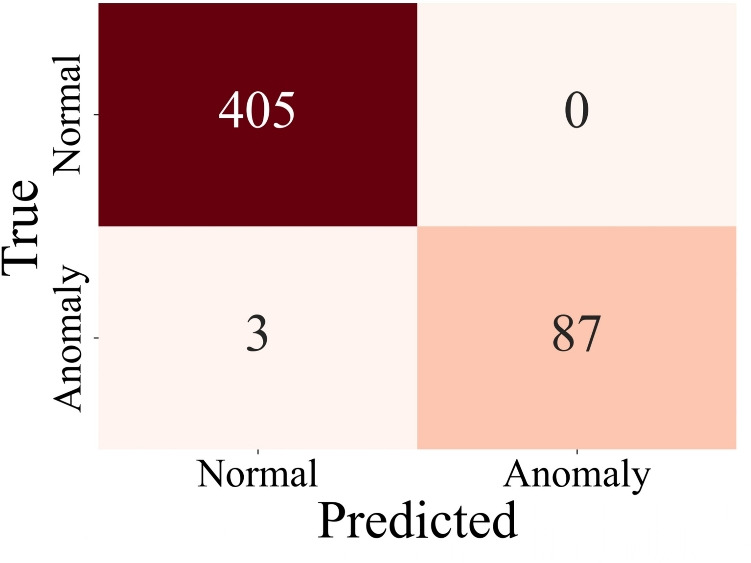
Adversal analysis confusion matrix.

Adversarially Trained Model Performance: After incorporating adversarial training, the model demonstrated exceptional robustness, achieving a adversarial accuracy of 99.39% and a error rate of 0.61%. All 405 normal samples were classified correctly, showing resilience against adversarial perturbations for benign data. The model correctly identified 87 out of 90 anomalous samples, with only 3 misclassifications (Fig. 10). Adversarial training effectively reduced the error rate by exposing the model to perturbed data during training, enabling it to learn the nuances of adversarial patterns. The adversarial accuracy increased from 95.15 to 99.39%, demonstrating a significant improvement in robustness.

The model’s performance on clean data remained unaffected, indicating that adversarial training does not compromise the detection of unperturbed samples. While the system was not explicitly tested against labeled zero-day scenarios, the adversarial training and evaluation using FGSM perturbations serve as a proxy for such cases. Adversarial inputs, although synthetically generated, help evaluate the model’s ability to generalize beyond its training distribution and reveal vulnerabilities to previously unseen behaviors. This analysis enhances confidence in the system’s robustness to emerging threats that may not yet be catalogued or formally modeled.

Adversarial training ensures the IDS is robust to adversarial attacks without sacrificing clean data performance. This capability is critical in operational environments, where malicious actors may exploit adversarial techniques to bypass anomaly detection systems. The FGSM for adversarial training as a starting point due to its computational efficiency and ease of implementation on edge-constrained hardware like the PYNQ-Z2.

To further evaluate the robustness of our proposed framework, we extended adversarial testing beyond the Fast Gradient Sign Method (FGSM) by implementing the iterative Projected Gradient Descent (PGD) attack. Unlike FGSM, which perturbs the input in a single step, PGD applies multiple iterative updates within an $\ell _\infty$-bounded $\epsilon$ ball, making it one of the most widely accepted and powerful first-order adversarial attacks. This provides a more rigorous assessment of the framework’s resilience under stronger adversarial scenarios.

In our experiments, adversarial examples were generated using varying perturbation budgets ($\epsilon = 0.02, 0.05, 0.10$) and step sizes proportional to $\epsilon$/steps. For each configuration, we evaluated both clean and adversarial accuracy, as well as the F1-score, to capture the effect of increasing perturbation strength on detection capability. The results are summarized in Table 6.

**Table 6:** Performance of LSTM under PGD adversarial attacks with varying perturbation budgets.

$\epsilon$	Steps	Clean Acc.	Clean F1	Adv.ersarial Acc.	Adversarial F1
0.02	10	0.912	0.834	0.912	0.834
0.05	20	0.901	0.811	0.901	0.811
0.10	30	0.851	0.754	0.851	0.754

The results demonstrate that the framework retains stable performance under PGD attacks, with minimal degradation observed compared to FGSM testing. Even at higher perturbation strengths ($\epsilon = 0.10$), the system maintained an adversarial accuracy of approximately 85.1% and F1-score of 75.4%. This indicates that the fusion of cyber-physical features and temporal modeling with LSTM provides inherent robustness against adaptive adversarial strategies. It is worth noting that while PGD is a significantly stronger attack than FGSM, the model’s resilience under these conditions suggests a capacity for generalization across diverse adversarial threat models.

### SHAP explainability

SHAP (SHapley Additive exPlanations) was used to interpret the predictions of the best-performing models. For the Random Forest model in multi-class classification, features such as eth_src, eth_dst, and frame_len were identified as the most influential. The key Observations were the eth_src exhibited strong interaction effects with nearly all other features. Frame_protocols and AL_Payload added contextual weight to predictions when combined with network identifiers.

SHAP reinforces the reliability of the model by offering transparent explanations, thus increasing trust in automated decisions. The results confirm that network communication patterns and content structure are critical in detecting cyber anomalies. While SHAP provides valuable insights into feature contributions and model decisions, it has limitations in robustness and interpretability under adversarial conditions. SHAP values explain what influenced the model’s prediction, but they do not guarantee the correctness or trustworthiness of the decision itself especially if the input has been adversarially perturbed. This limitation is inherent to post-hoc explainability methods.

### Hardware implementation

**Fig. 11:**
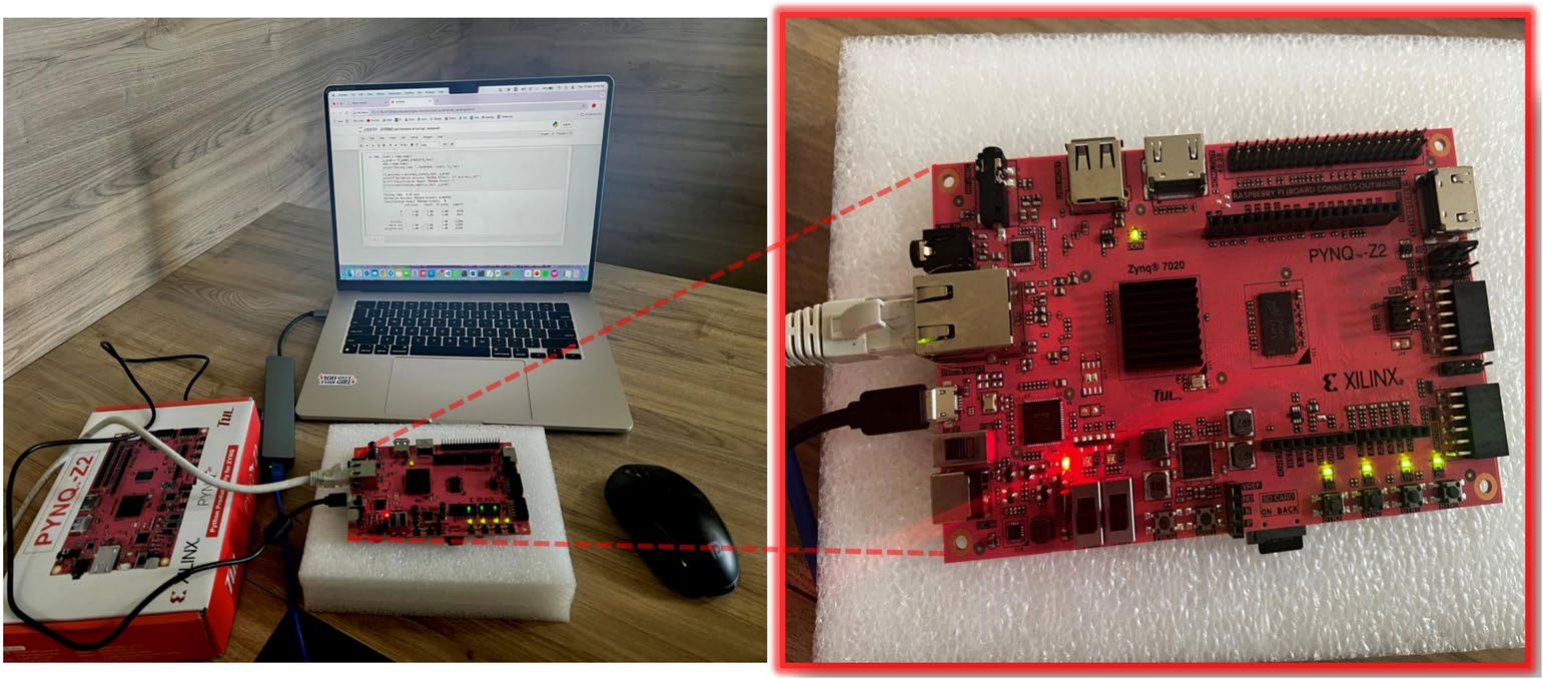
Hardware setup using PYNQ board.

An experimental evaluation was performed by implementing the Random Forest model on PYNQ based on the Xilinx Zynq board, an edge computing device^24,25^ to assess the feasibility of deploying our proposed model in real-world scenarios as shown in Fig. 11. The model’s performance was analyzed regarding its execution time for Purpose. It completed the training phase with a total duration of 82.56 seconds and a testing time of 2.16 seconds. The hardware setup and results demonstrated that the Random Forest model could operate on the edge device effectively, indicating its potential for real-time deployment in smart meter environments^26^. Using hardware-specific improvements like pipelining, parallelism and low-latency processing will speed up inference times and increase energy efficiency. The performance gains was further anticipated by tailoring the model architecture to FPGA constraints, making the deployment more feasible for large-scale, real-time smart metering applications. The use of Random Forest on the PYNQ-Z2 board aligns with prior studies on energy-efficient intrusion detection systems, where simpler models often yield favorable trade-offs between accuracy and resource usage^1^.

While LSTM yielded slightly higher classification accuracy in some cases, we selected Random Forest for edge deployment due to its lower computational footprint and faster inference speed. The PYNQ-Z2 board used in this study imposes strict hardware limitations, including limited memory and no dedicated GPU acceleration. Random Forest models are easier to quantize, require no floating-point operations, and can operate with reduced latency, making them ideal for real-time anomaly detection at the edge. This reflects a deliberate tradeoff between maximum accuracy and practical deployability, where our goal was to retain sufficient detection performance while enabling real-world integration.

## Conclusion

This work presents a comprehensive AI-driven cybersecurity framework designed for real-time anomaly detection in smart grid systems. By integrating cyber and physical data through advanced machine learning and deep learning techniques including Random Forests and Long Short-Term Memory (LSTM) networks, the system achieved exceptional performance in both binary and multi-class classification tasks. The use of SHAP explainability not only enhances transparency but also builds trust in automated decision-making processes. Notably, the framework’s adversarial training bolstered its resilience against evasion attacks, and successful deployment on edge hardware like the PYNQ-Z2 board affirms its potential for real-world, decentralized implementation in smart meters and substation environments. This framework can evolve by integrating automated threat response mechanisms to contain and recover from attacks without human intervention and adapting the model to other critical infrastructure sectors through transfer learning strategies.

## Supplementary Information

Supplementary Information.

## Data Availability

All data generated or analyzed during this study are included in this published article. The dataset used in our research was obtained from a publicly available repository on IEEE Dataport, originally contributed by Texas A&M University. Appropriate acknowledgment has been provided in the manuscript, with a detailed citation listed as Reference^11^ and further described in the Dataset Description section. The dataset can be accessed at: https://ieee-dataport.org/documents/cyber-physical-dataset-mitm-attacks-power-systems. The dataset used in this study is publicly available on IEEE DataPort: Cyber-Physical Dataset for MiTM Attacks in Power Systems (https://ieee-dataport.org/documents/cyber-physical-dataset-mitm-attacks-power-systems). It was generated at the RESLab testbed, Texas A&M University, USA, by executing various Man-in-the-Middle (MiTM) attacks on a synthetic electric grid. The testbed comprises a dynamic power system simulator (PowerWorld Dynamic Studio), a network emulator (CORE), Snort IDS, an open DNP3 master, and Elasticsearch’s Packetbeat index. The dataset includes both raw and processed files, enabling researchers and practitioners to extract new features or train intrusion detection systems (IDS) using the provided feature space.
